# How is becoming pregnant whilst HIV-positive? Voices of women at a selected rural clinic in Mpumalanga Province of South Africa

**DOI:** 10.1080/17290376.2020.1857299

**Published:** 2020-12-11

**Authors:** Livhuwani Muthelo, Judith Prudence Mgwenya, Rambelani Nancy Malema, Tebogo Mothiba

**Affiliations:** a Department of Nursing Science, University of Limpopo, Sovenga, South Africa; b Faculty of Health Sciences Executive Dean’s Office, University of Limpopo, Sovenga, South Africa

**Keywords:** Childbearing, human immunodeficiency virus, prevention of mother-to-child-transmission

## Abstract

**Background:**

Pregnancy decision-making is complicated for HIV-positive women because they have to contend with unpredictable symptoms, potential vertical transmission, and often a problematic life context including poverty, abuse, and stigma.

**Purpose:**

The purpose of the study was to explore the views of HIV-positive women attending a support group at a clinic in the Mpumalanga Province, on becoming pregnant.

**Methods:**

A qualitative, descriptive, and phenomenological research design was adopted to conduct one-on-one interviews using a semi-structured interview guide. Purposive sampling aided the selection of fifteen HIV-positive women who were members of the HIV/AIDS support group at the clinic. Data saturation was reached at participant number 15. Lincoln and Guba's four criteria for ensuring the trustworthiness of data were applied. Data were analysed using the open coding technique.

**Results:**

The following categories emerged: Mitigating fears of becoming pregnant through the prevention of mother-to-child transmission (PMTCT) programme; relationship between becoming pregnant and stigma attached to HIV/AIDS; cultural and social norms about becoming pregnant and the relationship between support groups and becoming pregnant.

**Conclusion:**

The study concluded that the desire to become pregnant amongst HIV-positive women is influenced by several aspects such as knowledge about the prevention of mother to child transmission, cultural values and social norms, and belonging to support groups where they were able to share experiences. Furthermore, becoming pregnant was viewed as an obligation to satisfy their partners/husbands and security to maintain marriages.

## 1. Introduction

Globally, there has been a 25% decline in new Human Immunodeficiency Virus (HIV) infections amongst young women between 2010 and 2018 (Global AIDS update, 2019). Although there has been a decline, there are still 6000 adolescent girls and young women infected every week (Global AIDS update, 2019). This is a worrying statistic in light of the fact that many countries committed themselves, in the 2016 United Nations Political Declaration, to ending Acquired Immune Deficiency Syndrome (AIDS) amongst adolescent girls and young women by 2020 (UNAIDS, 2016). It is estimated that in sub-Saharan Africa, three out of four new HIV infections are amongst adolescent girls (UNAIDS, 2019). In South Africa (SA), adolescent girls and young women are identified as the group with the highest rate of new HIV infections (George et al., 2020). Childbearing is considered of great importance in most parts of the world because it guarantees lineage continuity and offers hope for future economic emancipation (Chakare, 2013). In African communities ‘*Woman's glory is crowned in childbirth*’ (Baloyi, 2017; Van Zyl & Visser, 2015). Childless women are disrespected and abandoned by their partners and families, and in the process, also lose their ﬁnancial support (Baloyi, 2017; Van Zyl & Visser, 2015). Women are held more accountable for the survival of their marriages and are thus under more pressure to preserve the status quo (Baloyi, 2017; Chakare, 2013). In South Africa, the desire to become pregnant is high amongst both HIV-infected and HIV-uninfected women aged between 17 and 21 of whom the majority (67%) have not yet had children (Adler, Abar, Bennie, Sadeghi, & Bekker, 2017).

Pregnancy decision-making for HIV-positive women is complicated because women have to think about family planning, unpredictable symptoms and prognoses of the disease, the transmission of HIV to sexual partners, the risk of their children becoming orphans, unreasonable community expectations, and their problematic life context amidst poverty, substance abuse and stigma that may compromise their parenting abilities (Adler et al., 2017; Ivanova, Hart, Wagner, Aljassem, & Loufty, 2012). An increased incidence of maternal deaths has been documented in South Africa, with almost a third of deaths in 2015 estimated to be AIDS-related (Mnyani, Buchmann, Chersich, Frank, & McIntyre, 2017). However, Pillay (2019) documented that, with the modern medical treatment of HIV with antiretroviral therapy (ART), maternal death has drastically declined by 31% in 2017. Several variables including age, time since diagnosis of HIV, treatment with antiretroviral therapy (ART), and history of parity might influence pregnancy decisions amongst HIV-positive women (Adler et al., 2017).

Improved modern ART treatment includes the Option A and Option B approach. This initiative was recommended by the World Health Organization (WHO) in 2010 for Prevention of mother-to-child transmission (PMTCT). The Option A and Option B approach puts more emphasis on early screening for HIV suitability, longer enrolment for prophylaxis during the antenatal period and the provision of ART treatment during the lactation period (Chi, Stringer, & Moodley, 2013; WHO, 2010). From 2013, the majority of sub-Saharan Africa countries had rapidly shifted towards Option B+, which is a strategy in which all HIV-positive pregnant and lactating women are provided with lifelong antiretroviral treatment (ART) independent of CD4+ count (Cunnama et al., 2018; DiCarlo et al., 2019). A study by Chanda, Likwa, Zgambo, Tembo, and Jacobs (2018) exploring the acceptability of Option B+ among HIV positive women receiving antenatal and postnatal care services reported that HIV-positive women accepted lifelong treatment.

The desire to become pregnant can be determined by the knowledge and availability of professional counselling to explain the likelihood of becoming pregnant while being HIV-positive (da Silva, da Graça Corso da Motta, & Bellenzani, 2019). Based on the availability of PMTCT lifelong treatment, in the context of this study, the researchers explored the views of HIV-positive women who belonged to a support group about becoming pregnant. Therefore, it was against this background that the study explored the views of HIV-positive women attending a selected clinic in the Mpumalanga Province, South Africa with regards to becoming pregnant.

## 2. Methodology

### 2.1. Study design

A qualitative research approach applying phenomenological and descriptive designs was used to conduct the study. The phenomenological design enabled the researcher to gain an understanding of the participants’ perspectives (Rubin & Babbie, 2013) of their lived experiences regarding being HIV-positive and becoming pregnant, whereas the descriptive design enabled the researcher to obtain a picture of the natural situation as it happened (Burns & Grove, 2011).

### 2.2. Study setting

The study was conducted at a public Primary Health Care clinic in Mbombela, in the Ehlanzeni District of the Mpumalanga Province, South Africa. Mpumalanga lies to the north-east of South Africa and is South Africa's third most rural province. The Province is ranked second amongst the provinces of South Africa in terms of HIV prevalence. In 2014, the estimated Years of Life Lost (YLL) due to HIV and TB was 36.0 in Ehlanzeni, ranking the district amongst the worst in the country (MPAC, 2017). In 2014, the occurrence of HIV among pregnant women aged 15–49 years in the Ehlanzeni district of the Mpumalanga Province was (39.2%) (MPAC, 2017). The selected Primary Health Care clinic had two support groups for HIV-positive people that met once a month.

### 2.3. Population and sampling

The sample population was all HIV-positive women between the ages of 15 and 44 years. The target population was HIV-positive women who had been enrolled at the clinic for at least three months and had attended the support group at least three times (Grove, Gray, & Burns, 2015). A non-probability purposive sampling method was used to sample 15 HIV-positive women from the total population of 30 women.

### 2.4. Data collection procedure

Data were collected by the principal researcher from August to October 2014 through one-on-one interviews using an interview guide translated to Siswati, a language spoken by the majority of the people in the area. HIV-positive women were recruited from one of the support groups at the clinic. The details of the study, aims and objectives, and how the interviews were to be conducted were explained to the participants. Additionally, the researcher outlined that participation was voluntary and that they could withdraw at any time without fear of victimisation. An informed consent form was signed by each participant, after being informed of their rights as well as that the interview would be recorded, before data collection took place. Data were collected in a secluded room to maintain privacy. The central question posed to all participants was: **‘Can you describe your views on HIV-positive women concerning becoming pregnant?’** A voice recorder was used to capture verbal information and field notes were written to capture non-verbal cues. Data were collected until data saturation was reached at participant number 15.

### 2.5. Data analysis

The open coding data analysis method including an inductive, descriptive coding technique, as cited in Creswell (2014), was used to analyse the data. A list of all the themes was compiled and similar thoughts were clustered together and formed into columns, which were arranged into major themes, unique topics, and irrelevant issues, which were clustered separately. To establish whether new categories and codes had emerged, the themes were abbreviated as codes and were written next to the appropriate segments of the text. Irrelevant themes were discarded. Four categories and nine sub-categories finally emerged. An independent co-coder also analysed the data independently and a consensus with the researcher was later reached.

### 2.6. Trustworthiness

Trustworthiness was maintained by adhering to the criteria of credibility, confirmability, dependability, and transferability as outlined by Babbie and Mouton (2011). Credibility and confirmability were ensured by sending copies of the verbatim transcripts, voice recorded information, and field notes to an independent coder for analysis. The categories and sub-categories that emerged were compared with those of the researcher (De Vos, Strydom, Fouché, & Delport, 2012). Dependability and transferability were ensured through purposive sampling and providing a detailed description of the research methodology to assist researchers who want to replicate the study.

## 3. Ethical considerations

Ethical clearance was obtained from the University of Limpopo Medunsa Research and Ethics Committee (MREC/HS/291/2014) and permission to conduct the study was obtained from the Mbombela sub-district office and the Primary Health Care Manager. Participants were informed about the aim and objectives of the study and that participation in the study was voluntary: they were free to withdraw at any time, without any victimisation. Thereafter, all the participants who agreed to participate in the study were asked to sign a voluntary consent form.

## 4. Results

### 4.1. Participants demographic characteristics

Fifteen HIV-positive women were interviewed until data saturation was reached. The ages of the HIV-positive women ranged from 15 to 44 years. The majority of the participants were married with children whereas, others were either single or living with a partner. All the participants belonged to the support group at the selected clinic.

### 4.2. Categories and sub-categories reflecting the views of HIV-positive women on becoming pregnant

Four categories and eight sub-categories reflecting the views of HIV-positive women on becoming pregnant, emerged during data analysis. Each the categories will be presented with the relative sub-categories. Each sub-category will be supported by verbatim statements.

#### 4.2.1. Category 1: mitigating fears of becoming pregnant through the PMTCT programme

The rollout of the PMTCT of HIV and AIDS programmes in clinics has assisted in ensuring that pregnant women receive health education regarding available treatment during pregnancy and after delivery. Two sub-categories, namely, knowledge about the PMTCT programme and knowledge about AIDS and HIV increasing their compliance with treatment, emerged within this category and are discussed below.

##### 4.2.1.1. Sub-category 1.1: knowledge about PMTCT

The participants explained that they were not afraid of becoming pregnant because they had an understanding of the availability of treatment to prevent Mother-to-Child Transmission (MTCT) of HIV during pregnancy and after delivery. They also has knowledge about the treatment to be taken by the baby, exclusive breastfeeding and the use of condoms during sexual intercourse to prevent sexually transmitted infections. The following verbatim statements made by participants 8, 4, 1 and 2 respectively, reflected the extent and type of knowledge acquired by these HIV-positive women and how it had influenced their choice regarding becoming pregnant or not.

**Participant number 8:** ‘I am not scared to become pregnant because I am taking my treatment daily and when I get pregnant, I will continue taking the treatment to prevent my baby from being infected. It is also important to use a condom while pregnant to prevent the baby from getting HIV’.

**Participant number 4:** ‘It is important to start antenatal visits early so that if there is a problem it can be corrected before the damage. I have gained a lot by attending antenatal care in my first pregnancy. I tested positive on my first visit and did not know about HIV during that time, but every time I visit the clinic they would teach me. Through that, I managed to give birth to a healthy baby and I am also living a healthy life’.

**Participant number 1:** ‘If the mother chooses to breastfeed, she must give breast milk only and use a condom during sexual intercourse every time, because if they don't use a condom, the baby will get HIV from the breast milk’.

**Participant number 2 added by saying** ‘I don't have a child, but I know how to take care of myself when I get pregnant. The nurses teach us how to prevent HIV transmission from mother to baby. So the day when I am ready to have a baby, I will not struggle because I already know what to do and what to expect’.

##### 4.2.1.2. Sub-category 1.2: knowledge and understanding increases compliance

Having knowledge and understanding of how PMTCT works and the fact that the HIV-positive and AIDS infected participants’ health had improved after taking ARVs appeared to influence and motivate the participants to take ARVs. This is confirmed by the following verbatim responses:

**Participant number 9** said: ‘I was told to drink my ARVs at the same time every day and to avoid smoking and alcohol to live a better life. I feel better since I have started taking ARVs and I have been eating a healthy diet and I feel very fit that I can have another baby’.

**Participant number 4** added: ‘Every time my alarm rings, I know it's time to take my ARVs. If I get pregnant and get a baby, therefore I will also make the baby drink his/her medication at the same time because I got used to my schedule’.

#### 4.2.2. Category 2: relationship between becoming pregnant and the stigma attached to HIV/AIDS

Stigma, discrimination, and misconceptions about the disease by community members and the negative attitude towards people who are HIV-positive continued to be a hindrance towards disclosure of one's HIV status. Fear of disclosure might discourage the HIV-positive person from seeking treatment. Additionally, the participants said they have fear to disclose their status to their partners because in their village HIV-positive women are associated with prostitution. The following two sub-categories: fear of disclosure of their HIV status and the negative attitude of nurses towards patients with the above-mentioned statuses emerged from this category:

##### 4.2.2.1. Sub-category 2.1: fear of disclosing HIV status

From the verbatim statements, perceptions of what the community members and loved ones might say about the participant being HIV-positive, appeared to be responsible for the inculcation of the women's fear of disclosing their HIV-positive status.

**Participant number 13** said: ‘In my village when people hear that someone is HIV-positive, they stop interacting with that person. It becomes very difficult to tell your loved ones about your HIV status because they will reject you. That is why I have this fear of disclosing my HIV status. Some will say to you if someone becomes pregnant while being HIV-positive, that person is digging her own grave. It is worse when all these come from your loved ones’.

**Participant no. 7:** ‘I will never tell my husband about my HIV status because he always says people who get HIV are prostitutes. I asked him one time, what will happen if we both test positive. He said he will kill me and then kill himself. He wants more children and I agree but will never tell him about my HIV status’.

##### 4.2.2.2. Sub-category 2.2: negative attitude of nurses

**Participant number 13 and 14** reported that they were advised by the nurses in the selected clinic not to become pregnant because of their HIV status. They further explained that those who did not take their advice were scolded when they reported for antenatal visits.

**Participant number 13:** ‘The nurse shouted at me when I reported for antenatal care, she asked why did I become pregnant when I knew I am HIV positive. She further said I will be lucky if I do not die during delivery. I was so scared to return to the clinic, I even changed the clinics, here I am today being still alive and the baby is HIV negative’.

**Participant number 14:** ‘I heard nurses saying if you are HIV-positive you will get a miscarriage, or you will die during delivery or the baby can die. A lot of scary things were said about HIV and pregnancy, they make HIV look like a monster that kills people and it is not right, because I have a child and I am still healthy and alive’.

#### 4.2.3. Category 3: cultural and social norms about becoming pregnant

Having a baby was viewed as a gift from God, part of the culture, and a requirement in a marriage to satisfy the husband. The following three sub-categories emerged from this category: A child is a gift from God, satisfying your husband and marital obligations to have children and knowledge about contraceptives and the prevention of pregnancy.

##### 4.2.3.1. Sub-category 3.1: a child is a gift from God

A baby is a blessing from God, whether HIV-positive or negative. However, there was fear of becoming pregnant associated with the low CD4 count and adherence to the treatment.

**Participant no. 5**: ‘It was amazing when I held my baby for the first time, I thanked God for giving me such a wonderful gift. I knew my status before I fell pregnant and I was worried that I may not be able to have children. I am going to have the last one next year. I am not scared to have children because my CD4 count is very high, I take my treatment very well’.

**Participant number. 15 said**: ‘Having a child was the most amazing gift that God blessed my family with. Children are a gift from God it does not matter whether you are HIV-positive or not as long as you are taking treatment and eating well, you will have a healthy child’.

##### 4.2.3.2. Sub-category 3.2: satisfying your husband and marital obligations to have children

Becoming pregnant is a cultural and marital obligation to the entire family and the community as a whole. It was also viewed as a way to keep the marriage and prevention of polygamy in the marriage. More importantly, participants were pleased that though they were HIV-positive their children were HIV-negative.

**Participant no. 3 said:** ‘In my culture if you are married, you must make babies because if you fail to give your husband babies, he will either go out to have babies or will take a second wife. Having a child is the best gift you can give to keep your marriage. I have two kids and will get more if my husband asks for it. I have been HIV-positive since my first pregnancy and I am proud because all my kids are negative’.

**Participant no. 11 added**: ‘My husband is the only child in his family, giving him three kids was a blessing to the entire family. I discovered my HIV status during my second pregnancy and I have been on treatment since that day’.

**Participant number 14:** ‘It is very wrong to get pregnant when you are HIV-positive. Becoming pregnant cannot be a blessing from God if you are putting yourself and the baby at risk. A lot can happen during pregnancy and delivery. What if you die during pregnancy? Or your partner refuses to use a condom most of the time because the child can become positive. I think it is not right to get pregnant when you are HIV-positive’.

##### 4.2.3.3. Sub-category 3.3: knowledge about contraceptives and the prevention of pregnancy

The availability of different types of contraceptives to prevent pregnancy was suggested as a reason HIV-positive women were not becoming pregnant as often as they might, as indicated in the following quotations:

**Participant number 10 and 12 said**: ‘I don't see any reason why HIV-positive women should become pregnant when there are different types of injections available at the clinic. There is now a 3-year injection that prevents pregnancy. I will not become pregnant as long as there are those injections and condoms’.

It is important to use condoms to prevent sexually transmitted infections and to live a healthy life. Because if you don't use condoms, the CD4 count will go up and you will be weak and sick. If the mother chooses to breastfeed, she must give breast milk only and use a condom during sexual intercourse every time, because if they don't use a condom, the baby will get HIV from the breast milk.

#### 4.2.4. Category 4: the relationship between support groups and becoming pregnant

Many emotions confront women when they decide to bear children after they have been diagnosed with HIV. In this category, participants revealed that belonging to support groups taught them that they were not alone; by meeting people with similar experiences, they did not feel so alone and realised that there is an opportunity to build a new life. One sub-category: Belonging to support groups reduced stress, emerged from this sub-category.

##### 4.2.4.1. Sub-category 4.1: belonging to support groups alleviate fears and reduces stress

Being HIV-positive is stressful and belonging to a support group was viewed as being beneficial in terms of sharing fears and experiences related to becoming pregnant. Belonging to a support group also assisted HIV-positive women to have a sense of belonging when rejected by a community and/or loved ones.

**Participant number. 5**: ‘I could share with these people my deepest secrets and still be loved. Being around people with the same illness made me stronger. To me, they became family. We discuss all our challenges related to HIV and how to cope with the community and family members around you, more importantly, they emphasize the importance of taking treatment daily and eating healthy’.

**Participant number 8 added**: ‘Knowing that I wasn't alone made me realize that there is more to live for, than worrying about me being HIV-positive. They gave me a shoulder and were always there to listen when I felt rejected. Those who already become pregnant shared with us their experiences and it has helped me to understand that besides being HIV positive I can still have a healthy child’.

## 5. Discussion

Regardless of their HIV status, it is the desire of most woman to experience pregnancy, birth, and parenting (MacCarthy, Rasanathan, Ferguson, & Gruskin, 2012). The current study aimed to explore the views of HIV-positive women with regards to becoming pregnant. The study results revealed the decision of becoming pregnant amongst HIV-positive women is influenced by various factors that were classified according to categories: Mitigating fears of becoming pregnant through the PMTCT programme; The relationship between becoming pregnant and the stigma attached to HIV/AIDS; The relationship between support groups and becoming pregnant; and belonging to support groups alleviate fears and reduces stress.

### 5.1. Category 1: mitigating fears of becoming pregnant through the PMTCT programme

The study findings revealed that PMTCT is viewed by HIV-positive women as an important programme for HIV-positive women wanting to become pregnant. Participants outlined that the PMTCT programme is a source of knowledge regarding HIV/AIDS management during pregnancy and childbirth. The knowledge and availability had a positive influence on adherence to treatment and healthy living amongst HIV-positive women. Similarly, a qualitative study conducted in South Africa, Limpopo Province among HIV-infected mothers discovered that knowledge and awareness of MTCT and PMTC amongst HIV-positive mothers increased compliance with ARTs preventing Mother to Child Transmission (MTCT) (Ramoshaba & Sithole, 2017). Though the study did not explore the influence of Option B+ PMTCT on becoming pregnant, which was still new when the study was conducted, it would seem that the availability of the life-long anti-retroviral treatment, regardless of CD4 count, could have influenced the desire to become pregnant. A study by Matheson et al. (2015) documented that, globally, HIV-positive women are aware that the introduction of Option B+ PMTCT has health benefits for both the mother and the baby. In Tanzania, a study done by Haile, Tewoldeberhan, and Chertok (2016) indicated that HIV-positive women have more information about mother-to-child transmission (MTCT) of HIV and its prevention than HIV-negative women.

### 5.2. Category 2: the relationship between becoming pregnant and the stigma attached to HIV/AIDS

The HIV-positive women expressed that there is still stigma and discrimination attached to being HIV-positive and having AIDS. They raised the concern that they fear disclosing their HIV status to their loved ones because of the stigma attached to HIV within the community as a whole. They also revealed that in their community, women who are HIV-positive are associated with prostitution. This has also been confirmed by a study done in Canada Ivanova et al. (2012) and a review paper by Carlsson-Lalloo, Rusner, Mellgren, and Berg (2016), in which they also reported that the stigma attached to HIV/AIDS is a barrier to the disclosure of HIV status amongst HIV-positive women, which could consequently lead to rejection and isolation. Stigma and fear affects HIV-positive individuals psychologically, resulting in relationship problems and family distress.

The study findings have shown that HIV-positive women alleged that the nurses in some clinics displayed negative attitudes towards them. HIV-positive women who tried to seek advice were scolded. This finding is in conflict with the new South African Department of Health's approach of strengthening PMTCT programmes, which was achieved by introducing new guidelines and training of health care workers (NDOH, 2015). Another study conducted in Durban (SA) investigating knowledge and attitudes of HIV infection and PMTCT revealed that 13% of the respondents had been treated badly by health workers because of their HIV-positive status. The same study reported an element of insensitivity amongst doctors and nurses towards HIV infected people, which contributed to patients losing hope in public hospitals (Haffejee, Ports, & Mosavel, 2016).

### 5.3. Category 3: cultural and social norms about becoming pregnant

The desire to bear a child is influenced by cultural and social norms. Irrespective of HIV status, socially as a woman, bearing children is essential to fulfilling the marital expectations of both the family and the husband/partner. In this study, HIV-positive women reported that they wished to become pregnant to preserve the marriage. More importantly, a child is seen as a blessing to the couple and their extended family, as well as a gift from God. A similar study in Rakai (Uganda) indicated that most HIV-positive women reported that it was their spouse's wish to have children and not necessarily their own (Makumbi et al., 2011). Studies by Carlsson-Lalloo et al. (2016) and Behboodi-Moghadam, Khalajinia, Nasrabadi, Mohraz, and Gharacheh (2015) have indicated that many women expressed the perception that having a baby was a gift from God and something magical. They further indicated that they passed the responsibility on to God to protect them.

### 5.4. Category 4: the relationship between support groups and becoming pregnant

Through the support obtained from support groups, participants explained that their fears of becoming pregnant were alleviated. Belonging to support groups had helped them cope with the stress experienced after being diagnosed as HIV-positive. Through the support groups, they had learned that they were not alone; meeting people with similar experiences made them less self-aware and helped them realise that there is an opportunity to build a new life. Belonging to a support group also had a remarkable impact on reducing morbidity and stress (Bateganya, Amanyeiwe, Roxo, & Dong, 2015). Being a member of a support group has the potential benefit of compliance with ART treatment and improved quality of life. This is achieved through the members of the support sharing coping skills on how to live with being HIV-positive.

Specific positive benefits associated with support group membership included enhancing treatment adherence success and improving the quality of life through equipping HIV-positive people with coping skills (Kim, Gerver, Fidler, & Ward, 2014). In addition, Mundell et al. (2011) observed that the lives of participants who belonged to support groups changed positively and they also displayed higher levels of self-esteem. More importantly, support group's enhanced disclosure of HIV-positive status with the potential benefit of prevention of re-infection (Bateganya et al., 2015).

## 6. Limitations of the study

Only HIV-positive women who had been enrolled at the clinic for three months and belonging to the support group were included in this study. HIV-positive women within a support group are more likely to be aware of the programmes and options available to assist them in becoming pregnant. The study was limited to only one clinic and the sample size was small. The results can therefore not be generalised beyond the study participants.

## 7. Conclusion

In this study, the participants demonstrated that they had sufficient knowledge about PMTCT. The extent and variety of knowledge about PMTCT alleviated their fears of their becoming pregnant because they were aware that the chance of transmitting HIV to their babies was limited. The culture of having children in marriage had influenced some women to have children. There were, however, those who feared becoming pregnant due to the stigma still attached to being HIV-positive. The negative attitude of healthcare providers towards pregnant women who were HIV-positive also discouraged some women from becoming pregnant for fear of being scolded. Negativity towards people who are HIV-positive led some of them not to disclose their HIV status to their partners. In conclusion, those who belonged to a support group felt supported and empowered.
